# Plasma-Based Longitudinal Evaluation of *ESR1* Epigenetic Status in Hormone Receptor-Positive HER2-Negative Metastatic Breast Cancer

**DOI:** 10.3389/fonc.2020.550185

**Published:** 2020-09-18

**Authors:** Lorenzo Gerratana, Debora Basile, Alessandra Franzoni, Lorenzo Allegri, Davide Viotto, Carla Corvaja, Lucia Bortot, Elisa Bertoli, Silvia Buriolla, Giada Targato, Lucia Da Ros, Stefania Russo, Marta Bonotto, Barbara Belletti, Gustavo Baldassarre, Giuseppe Damante, Fabio Puglisi

**Affiliations:** ^1^Department of Medicine (DAME), University of Udine, Udine, Italy; ^2^Department of Medical Oncology, Centro di Riferimento Oncologico di Aviano (CRO), IRCCS, Aviano, Italy; ^3^Institute of Human Genetics, ASUFC University Hospital, Udine, Italy; ^4^Unit of Molecular Oncology, Centro di Riferimento Oncologico di Aviano (CRO), IRCCS, Aviano, Italy; ^5^Department of Oncology, ASUFC University Hospital, Udine, Italy

**Keywords:** circulating tumor DNA, DNA methylation, endocrine treatment, ESR1, liquid biopsy

## Abstract

**Background:**

Endocrine therapy (ET) is the mainstay of treatment for hormone receptor-positive human epidermal growth factor receptor 2 (HER2)-negative metastatic breast cancer; however, adaptive mechanisms emerge in about 25–30% of cases through alterations in the estrogen receptor ligand-binding domain, with a consequent ligand-independent estrogen receptor activity. Epigenetic-mediated events are less known and potentially involved in alternative mechanisms of resistance. The aim of this study was to test the feasibility of *estrogen receptor 1* (*ESR1*) epigenetic characterization through liquid biopsy and to show its potential longitudinal application for an early ET sensitivity assessment.

**Methods:**

A cohort of 49 women with hormone receptor-positive HER2-negative MBC was prospectively enrolled and characterized through circulating tumor DNA using methylation-specific droplet digital PCR (MS-ddPCR) before treatment start (BL) and after 3 months concomitantly with computed tomography (CT) scan restaging (EV1). *ESR1* epigenetic status was defined by assessing the methylation of its main promoters (promA and promB). The most established cell-free tumor DNA (ctDNA) factors associated with ET resistance [*ESR1* and phosphatidylinositol-4,5-bisphosphate 3-kinase, catalytic subunit alpha (*PIK3CA*) mutations] were assessed through next-generation sequencing. Associations were tested through Mann–Whitney *U* test, matched pairs variations through Wilcoxon signed rank test, and survival was analyzed by log-rank test.

**Results:**

The ET backbone was mainly based on aromatase inhibitors (AIs) (70.83%) in association with CDK4/6 inhibitors (93.75%). Significantly lower promA levels at baseline were observed in patients with liver metastases (*P* = 0.0212) and in patients with *ESR1* mutations (*P* = 0.0091). No significant impact on PFS was observed for promA (*P* = 0.3777) and promB (*P* = 0.7455) dichotomized at the median while a ≥2-fold increase in promB or in either promA or promB at EV1 resulted in a significantly worse prognosis (respectively *P* = 0.0189, *P* = 0.0294). A significant increase at EV1 was observed for promB among patients with *PIK3CA* mutation (*P* = 0.0173). A trend was observed for promB in *ESR1* wild-type patients and for promA in the *ESR1* mutant subgroup.

**Conclusion:**

The study proofed the concept of an epigenetic characterization strategy based on ctDNA and is capable of being integrated in the current clinical workflow to give useful insights on treatment sensitivity.

## Background

Breast cancer (BC) is a complex disease encompassing clinically and molecularly heterogeneous tumors. Approximately 70% of BC express hormone receptors and are, therefore, eligible for endocrine treatment (ET). In the last decades, several ETs have been introduced in the management of hormone receptor-positive human epidermal growth factor receptor 2 (HER2)-negative (luminal-like) BC, including aromatase inhibitors (AIs), selective estrogen receptor modulators (SERM), and degraders (SERD), that dramatically decreased mortality (1–3). Despite that, about 25–30% of metastatic BC (MBC) previously treated with ET develop resistance due to alterations in the estrogen receptor (ER) ligand-binding domain, showing a ligand-independent ER activity and in particular, up to 20% develop mutations in the ER gene (*ESR1*) (4–7). Although the main known ET resistance mechanisms rely on the mutations of few genes, they account only for 40% of cases, as genetic-mediated events are not the only mechanisms capable to perturbate *ESR1* activity and expression (8, 9). Despite being initially neglected, *ESR1* mutations are currently one of the main known ET resistance factors in luminal-like MBC. As a matter of fact, *ESR1* mutations are not often present in primary tumors but are rather selected during AI-based therapies and eventually characterize the dominant clone when disease progression occurs (10). Moreover, their onset is associated with a lower treatment benefit in subsequent lines when an AI-based backbone is selected, while discordant data are available with respect to SERDS (5, 11). DNA methylation, an epigenetic phenomenon, leads to gene silencing through the addition of a methyl group to the fifth carbon of the cytosine residue in the context of GcP islands (CGIs) and *cis*-regulatory elements (CREs), as promoter and enhancer regions (12–15). In luminal-like MBC, the *ESR1* promoter methylation drives the silencing of *ESR1*, with a loss of ER tissue expression and consequent ET resistance (16). *ESR1* is located in an extremely complex locus of 450 kb in chromosome 6q25.1 and its expression is regulated by several promoters. The different transcripts generated by each promoter show a unique 5’-untranslated region, and they are subject to splicing to form a single mRNA (17). Several promoters are involved in *ESR1*’s tissue-specific regulation and in particular, two proximal promoters (promoter A and B) are located within ∼2 kb of the transcription start site and are transcriptionally active in BC (18–20).

Liquid biopsy, based on the analysis of circulating cell-free tumor DNA (ctDNA) and circulating tumor cells (CTCs), is gaining momentum as a noninvasive real-time tumor-monitoring tool that may reflect tumor biology and evolution. This concept was explored by analyzing the most established ET resistance factors, *ESR1* and phosphatidylinositol-4,5-bisphosphate 3-kinase, catalytic subunit alpha (*PIK3CA*) mutations, in the PALOMA-3 study. While it has been highlighted that a relative change in *PIK3CA* allele frequency after 15 days strongly predicted PFS on palbociclib and fulvestrant, on the other hand, *ESR1* dynamics offered limited information on the long-term clinical outcome, probably due to early divergent response of tumor subclones to treatment and the more gradual onset of new *ESR1* mutations (21). On the other hand, few studies have proved the association between ET sensitivity and *ESR1* promoter methylation (22).

The aim of this study was to test the feasibility of *ESR1* epigenetic characterization through liquid biopsy and to show its potential longitudinal application for an early ET sensitivity assessment.

## Materials and Methods

### Study Population and Ethics Statement

A cohort of 49 women with luminal-like MBC was prospectively enrolled in the CRO-2018-56 multicenter pragmatic study, between 2018 and 2019. All patients were diagnosed with luminal-like MBC and received either fulvestrant or AIs with or without CDK4/6 inhibitors as first-line ET according to the investigator’s choice. Diagnosis of any secondary malignancy within the last 3 years and prior ET for MBC were the two main exclusion criteria. Patients could have received both ET and chemotherapy in the adjuvant and neoadjuvant setting. Samples were collected before treatment start [baseline (BL)] and after 3 months concomitantly with computed tomography (CT) scan restaging [first evaluation (EV1)]. The study was approved by the ethics committee under the CEUR-2018-Sper-056-CRO protocol.

### Extraction of Circulating Tumor DNA From Plasma Samples

Blood samples were collected using the PAXgene Blood ccfDNA Tubes (Qiagen) or the Cell-Free DNA BCT tubes (Streck). Plasma was then recovered and stored at −80°C. ctDNA was isolated from 4.8 ml aliquots of plasma with the QIAsymphony PAXgene Blood ccfDNA Kit (Qiagen) through the QIAsymphony SP instrument (Qiagen) using the recommended Standard Protocol Line (STA) for small fragment enrichment and eluted in 60 μl of elution buffer (Qiagen). ctDNA concentration was estimated using the Qubit 1X dsDNA HS Assay Kit (Qiagen).

### Next-Generation Sequencing

Primers (Sigma) were designed to amplify the regions of the genes that contained hotspot mutation and were built with different 5′-adapter region. To prepare the next-generation sequencing (NGS) library, we carried out two consecutive rounds of PCR, both of which used Phusion Hot Start II High-Fidelity DNA Polymerase (Thermo Fisher). In the first round, primers recognized and amplified the region(s) of interest, and in the second one, PCR products from first round were diluted 1:100 and then amplified with Index primers (IDT) that barcoded each sample for subsequent univocal identification. After amplification, Wizard^®^ SV Gel and PCR Clean-Up System (Promega) were used to purify the fragments, following the manufacturer’s instructions. Finally, all samples were diluted to a final concentration of 2 nM, to create the library. Phix sequencing Control (Illumina) was added and the library was finally denatured and loaded into the cartridge (Illumina).

Sequencing data were exported as BAM and VCF files and analyzed using the IGV software and the VariantStudio software (Illumina), respectively (Supplementary Data Sheet 1). After quality check controls and filtering, reads and variants distributed in target regions were analyzed and annotated.

### Methylation-Specific Droplet Digital PCR

CpG island prediction and primer/probe design were assessed on the Methprimer 2.0 online platform according to standard recommendations (23). Probes’ sequences included at least three potential methylation sites, and probe-specific binding sites included several cytosines, ensuring specificity for converted DNA. No CpG were included in primer pairs to enforce methylation-independent primer efficiencies. The amplicon size was kept on 100 bp or less, to efficiently anneal on ctDNA fragments. Two probes were designed: one was labeled in 6-FAM and was specific for methylated DNA and the other was labeled in HEX specific for un-methylated DNA.

The EZ DNA Methylation-Gold^TM^ Kit (Zymo Research) was used for bisulfite conversion of 10 ng ctDNA, according to the manufacturer’s standard protocol. Bisulfite-converted ctDNA was eluted in 20 μl elution buffer for downstream analysis. Ten nanograms of Human WGA Methylated and Non-methylated DNA Sets (Zymo Research) were bisulfite converted and processed with each droplet digital PCR (ddPCR) experiment as positive and negative controls.

Five microliters of bisulfite-converted ctDNA were then used for absolute quantification of *ESR1* promoter methylation levels. Samples were considered positive for methylation when at least three positive droplets were evaluated. The total number of copies/reactions was assessed, and methylation levels were expressed as fractional abundance of methylated DNA alleles [beta value (β)]. Samples were run in duplicated and then merged for further analysis. Droplets were generated on the automated droplet generator QX200 AutoDG^TM^ (Bio-Rad), and after PCR amplification, droplets were read on the QX200^TM^ Droplet Reader (Bio-Rad). Data were analyzed using the QuantaSoft^TM^ 1.7.4 Software (Bio-Rad).

### Statistical Analysis

Clinico-pathological characteristics were summarized through descriptive analysis. Categorical variables were described through frequency distribution, whereas continuous variables were reported through median and interquartile range (IQR). The BL staging was performed concomitantly to liquid biopsy. Distant localizations were categorized based on the presence of specific organ involvement (e.g., liver involvement, yes vs. no) independently from other metastatic sites.

Associations between promoter methylation, clinico-pathological characteristics, and detectable mutations were explored through Mann–Whitney *U* test. The association between metastatic sites and median level of promA/B was investigated through uni- and multivariate logistic regression. Matched pairs variations across BL and EV1 were tested through Wilcoxon signed rank test.

Progression-free survival (PFS) was defined as the time from study enrollment to disease progression (according to RECIST criteria) or death from any cause or date of last follow-up, while overall survival (OS) was defined as the time from study enrollment to death from any cause. Censoring was applied to patients without an endpoint event at the last follow-up visit. Survival was represented by Kaplan-Meier estimator plot and analyzed by log-rank test. The differential prognostic impact of promA/B across clinically relevant subgroups was tested by Cox regression and represented though forest plot.

All *P*-values were two sided, with *P* < 0.05 considered the threshold for statistical significance.

Statistical analysis was conducted using the StataCorp 2016 Stata Statistical Software: Release 15.1 (College Station, TX, United States), R (The R foundation for Statistical Computing. version 3.3.1) (2016-06-21), and JMP (SAS Institute, version 14).

## Results

A cohort of 49 patients with luminal-like MBC treated with first-line ET was prospectively enrolled between May 2018 and November 2019. One patient rapidly progressed during the induction of ovarian suppression and was therefore not eligible for the analysis at EV1. The first-line ET backbone was mainly based on AIs (70.83%) in association with CDK4/6 inhibitors (93.75%). Of the 11 patients treated with a fulvestrant-based ET, 9 patients relapsed during adjuvant ET. Among them, five relapsed during the extended adjuvant phased (i.e., after more than 5 years of adjuvant treatment).

Consistently with literature, bone metastases were the most represented (Table 1) (24, 25). Median follow-up was 11.9 months (IQR, 10.1–19.3).

**TABLE 1:** Clinico-pathological characteristics of the study cohort.

	Median	IQR
Age (years)	63	52–71
ER (%)	95	90–95
PR (%)	50	20–75

	***N***	**%**

**Age**		
<50	10	20
≥50	39	80
***ESR1* status**		
Wild type	40	82
Mutated	7	14
Not evaluable	2	4
***PIK3CA* status**		
Wild type	36	72
Mutated	11	22
Not evaluable	2	4
**Liver involvement**		
No	35	73
Yes	14	27
**Lung involvement**		
No	38	78
Yes	11	22
**CNS involvement**		
No	49	100
Yes	0	0
**Bone involvement**		
No	17	35
Yes	32	65
**Lymph nodes involvement**		
No	32	65
Yes	17	35
**Soft tissue involvement**		
No	45	92
Yes	4	8
**First-line treatment agents**		
AI single agent	1	2
Fulvestrant single agent	2	4
AI and CDK4/6i	34	71
Fulvestrant and CDK4/6i	11	23

**IQR*, interquartile range; *ER*, estrogen receptor; *PR*, progesterone receptor; *ESR1*, estrogen receptor 1; *PIK3CA*, phosphatidylinositol-4,5-bisphosphate 3-kinase, catalytic subunit alpha.*

*ESR1* and *PIK3CA* mutations are currently the most established ctDNA factors associated with ET resistance and were therefore assessed through an amplicon-based NGS panel for a more complete baseline characterization.

*ESR1* and *PIK3CA* were mutated in 7 (14%) and 11 (23%) patients, respectively. *ESR1* variants were Y537S (3/7, 44%), Y537N (1/7, 14%), H377R (1/7, 14%), and D538G (2/7, 28%), while *PIK3CA* variants were H1047R (8/11, 73%), E545K (2/11, 18%), and H1047L (1/11, 9%). Full details are available in Supplementary Table 1.

**FIGURE 1:**
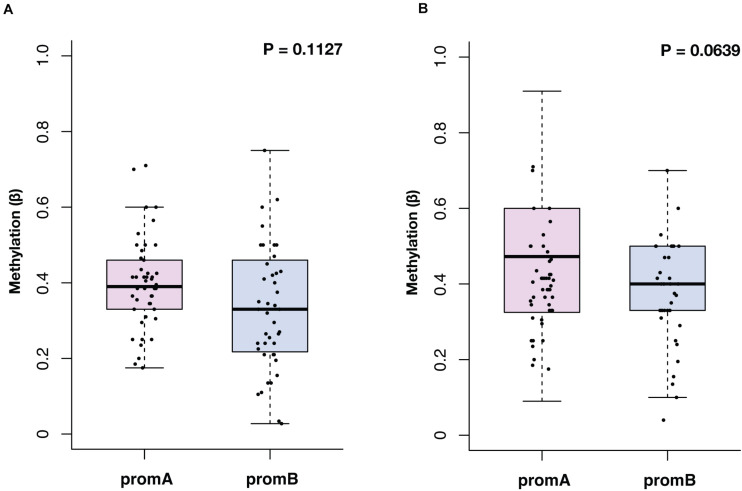
Overall distribution of *ESR1* promoter A (promA, purple) and B (promB, blue) methylation at baseline **(A)** and at first CT scan evaluation after 3 months of ET **(B)**. Median methylation at baseline was 0.39 for promA and 0.33 for promB (*P* = 0.1127) **(A)**. At first CT scan evaluation after 3 months of ET median methylation was 0.47 for promA and 0.40 for promB (*P* = 0.0639) **(B)**.

The median methylation at BL for promoter A (promA) was 0.39 (IQR, 0.33–0.43) and 0.33 (IQR, 0.22–0.46) for promB, with no significant differences in the overall population (*P* = 0.1127) (Figure 1A). At EV1, promA and promB medians were, respectively, 0.47 (IQR, 0.33–0.60) and 0.40 (IQR, 0.33–0.50) at EV1 (*P* = 0.0639) (Figure 1B). No direct correlation was observed after Shapiro–Wilk normality testing at BL (*R*^2^ = 0.0978) and EV1 (*R*^2^ = 0.0265).

### Baseline promA Varied Across Baseline Characteristics and Was Not Associated With Prognosis

**FIGURE 2:**
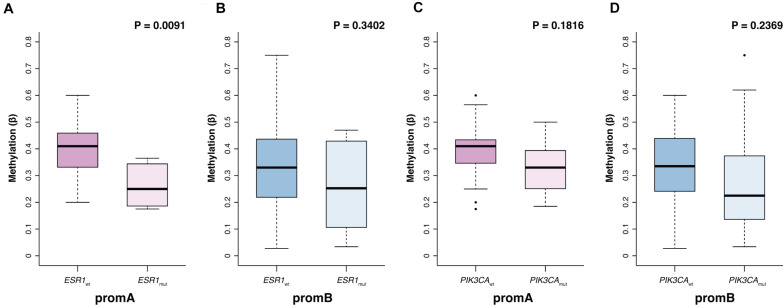
Promoter A and B methylation (promA and promB) according to *ESR1* and *PIK3CA* mutational status. PromA methylation levels were lower in patients with a ctDNA-detectable *ESR1* mutation **(A)**. No associations were observed with respect to PIK3CA mutational status or in ESR1 wild type patients **(B–D)**.

Promoter A and B were investigated at BL to explore potential factors associated with *ESR1’s* epigenetic status (Table 2). No differences were observed with respect to age, while significantly lower promA levels were observed in patients with liver metastases (*P* = 0.011). No significant variations were observed across all investigated sites for promB, numerically higher promB levels were detected in patients with soft tissue involvement (Table 2). PromA was significantly lower in patients with ESR1 mutations (median, 0.41; IQR, 0.33–0.46; median, 0.25; IQR, 0.19 0.35, respectively, in ESR1 wild-type and mutated; *P* = 0.0091) (Figure 2A). No associations were observed with respect to PIK3CA mutational status (Figure 2).

**TABLE 2:** Distribution of promoter A and B methylation levels (promA and promB) across age groups and metastatic sites.

	promA			promB		
		
	Median	IQR	*P*	Median	IQR	*P*
**Age**						
<50	39	28–44.25	0.7213	32	21–50	0.8304
≥50	38.5	33–46.5		33	21.8–46	
**Liver involvement**						
No	41.5	34.5–50	0.0196	33.75	21–48.5	0.5009
Yes	36.5	25–38.5		28	23.3–42	
**Lung involvement**						
No	41.3	33.8–48.3	0.1498	32	21–45	0.2486
Yes	36.5	30.5–40.5		40	24–50	
**Bone involvement**						
No	41.5	36.5–46	0.1268	37.3	30.8–50	0.1017
Yes	38.5	31–41.5		26.8	20.3–44	
**Lymph nodes involvement**						
No	38.5	33–43.5	0.9625	32	21–42.5	0.2150
Yes	39.5	31–48.5		37.5	24–50	
**Soft tissue involvement**						
No	40.5	33–46.5	0.1137	32	21–42.8	0.0688
Yes	31	30.5–35.5		50	38.3–55	

*Methylation levels were significantly lower for promA in patients with liver involvement (*P* = 0.0196). Significance was tested through the Mann–Whitney *U* test.*

The association between metastatic sites and promA/B was further explored through multivariate logistic regression after dichotomization at the median value. The inverse association between promA and liver involvement was confirmed (OR = 0.09; 95% CI = 0.01–0.58; *P* = 0.0109) (Table 3).

**TABLE 3:** A significantly lower promA was confirmed in patients with liver involvement after correction for the most represented metastatic sites (*P* = 0.0109).

	promA			promA		
		
	OR	95% Cl	*P*	OR	95% Cl	*P*
**Liver involvement**						
No	1		0.0109	1		0.1745
Yes	0.09	0.01–0.58		0.34	0.07–1.61	
**Lung involvement**						
No	1		0.0635	1		0.7761
Yes	0.14	0.02–1.12		1.28	0.23–7.06	
**Bone involvement**						
No	1		0.1118	1		0.1711
Yes	0.28	0.06–1.35		0.33	0.07–1.61	
**Lymph nodes involvement**						
No	1		0.0806	1		0.3078
Yes	5.40	0.81–35.80		2.43	0.44–13.44	

*Significance was tested through multivariate logistic regression, promA and B were dichotomized at median.*

In this subgroup, median ER expression of the most updated biopsy was 90% (range, 30–100%) while the median ER expression was 95% (range, 80–100%) in patients without liver involvement.

**FIGURE 3:**
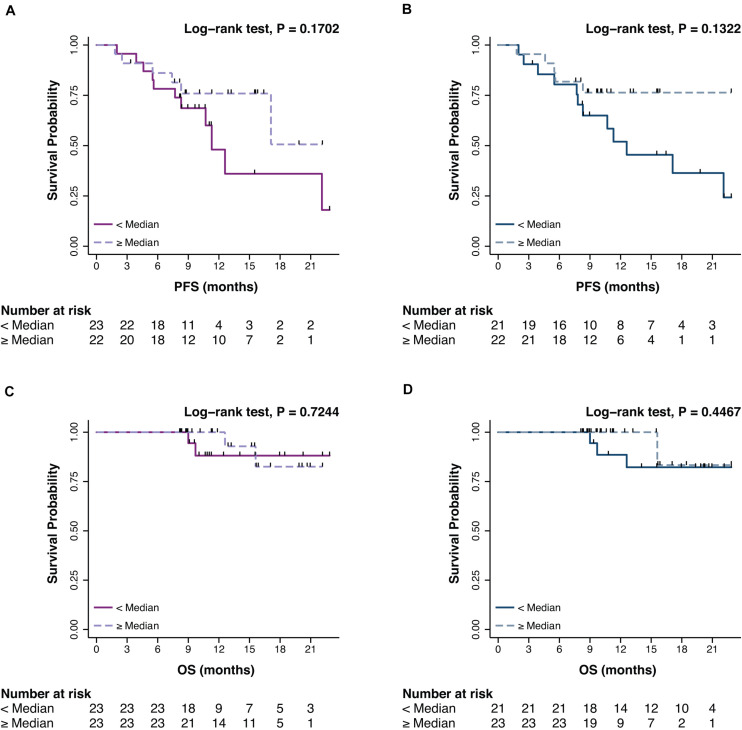
Impact on PFS and OS of promA (purple) **(A,C)** and promB (blue) **(B,D)**. No significant impact was observed for promA and promB dichotomized at the median for both PFS (respectively *P* = 0.1702 and *P* = 0.1322) **(A,B)** and OS (respectively *P* = 0.7244 and *P* = 0.4467) **(C,D)**.

The prognostic impact of promA and promB was then explored in terms of PFS and OS. At BL, no significant impact was observed for promA and promB dichotomized at the median for both PFS (respectively *P* = 0.1702 and *P* = 0.1322) (Figures 3A,B) and OS (respectively *P* = 0.7244 and *P* = 0.4467) (Figures 3C,D).

### The Longitudinal Evaluation of prom A/B Is Feasible and Can Give Insights on Treatment Sensitivity

**FIGURE 4:**
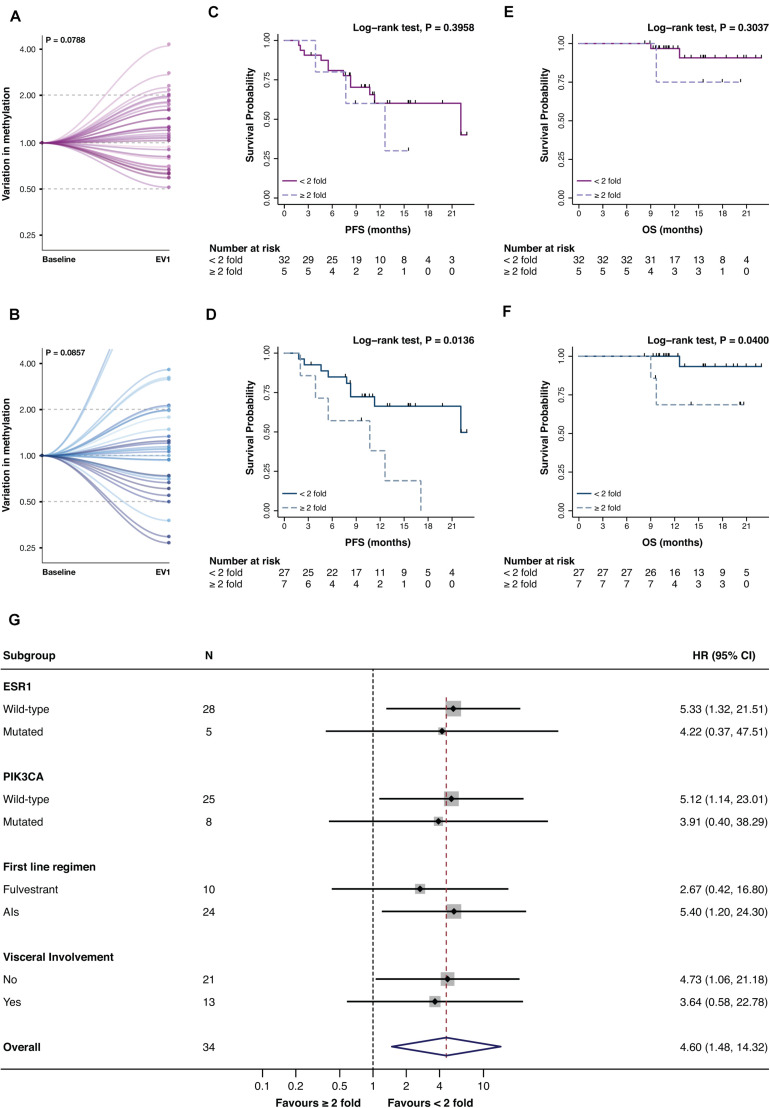
Variation of promA (purple) and promB (blue) between BL and EV1 **(A,B)** and impact on PFS **(C,D)** and OS **(E,F)** of a ≥2-fold increase in promA and promB. Subgroup analysis of the impact on PFS of a ≥2-fold increase in promB **(G)**. A numerical increase in promA was observed after normalization to the baseline levels. For two cases, an 18- and 15-fold increase in promB was observed **(B)** and were, therefore, out of scale **(B)**. A ≥2-fold increase in promA was not associated with PFS or OS **(C,E)**, while a doubling in promB resulted in a significantly worse prognosis both on PFS and OS **(D,F)**. Notably, subgroup analysis showed a consistent impact on PFS across different ET backbones and according to *ESR1* and *PIK3CA* mutational status.

Promoter A and B were then assessed at EV1 (Figure 1B). Only numerical variations were observed from BL to EV1 for promA and promB after normalization on the BL values (respectively *P* = 0.0788 and *P* = 0.0857), notably two cases showed a 15- and 18-fold increase in promB levels (Figures 4A,B).

The impact of a variation in promA and promB between BL and EV1 was investigated both in terms of PFS and OS. To exclude possible biasses derived from the biological meaning of undetectable ctDNA levels, only patents with detectable methylation at BL and EV1 were included in this analysis (26).

While promA doubling alone has no impact on PFS nor OS (Figures 4C,E), a ≥2-fold increase in promB resulted in a significantly worse prognosis both in terms of PFS and OS (respectively *P* = 0.0136 and *P* = 0.0400) (Figure 4D,F). The prognostic impact of a 2-fold increase in promB was explored across clinically relevant subgroups. In the total population, hazard ratio (HR) was 3.47 (95% CI, 1.20–10.04; *P* = 0.0214), consistent results were observed according to visceral involvement, first-line ET backbone, and *ESR1* and *PIK3CA* mutational status (Figure 4G).

**FIGURE 5:**
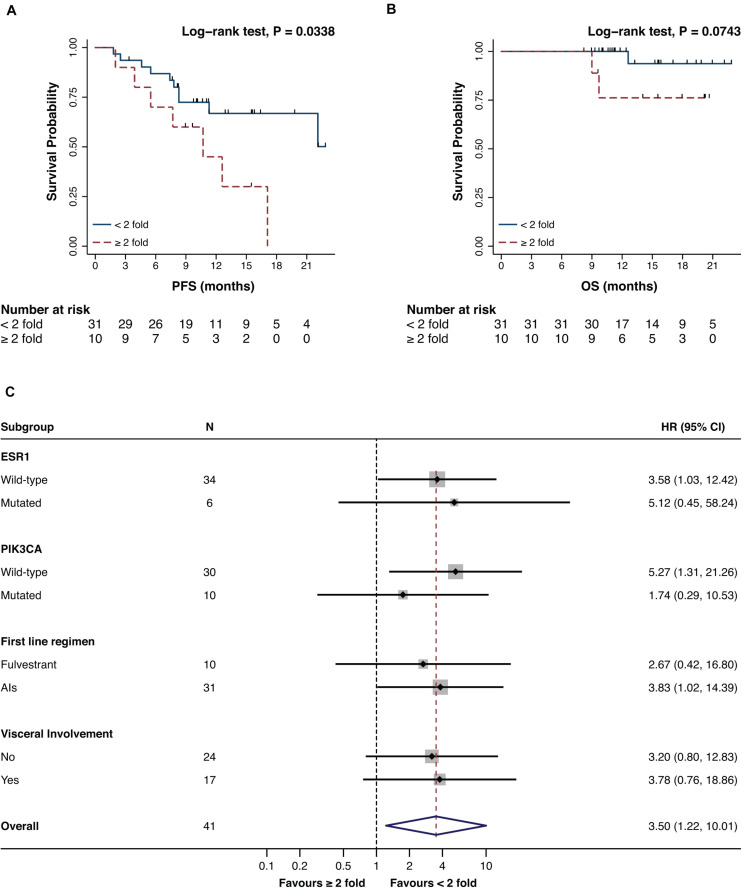
Impact on PFS **(A)** and OS **(B)** and subgroup analysis in terms of PFS **(C)** of a ≥2-fold increase in either promA or promB. A ≥2-fold increase in promA or promB was associated with an impact on PFS **(A)**. Subgroup analysis showed a consistent impact on PFS across different ET backbones and according to *ESR1* and *PIK3CA* mutational status **(C)**.

The prognostic impact of a 2-fold increase of either promA or promB was moreover investigated. A ≥2-fold increase in either promA or promB was associated to a significantly worse prognosis in terms of PFS (*P* = 0.0338) (Figures 5A,B) that was consistent across all considered subgroups (Figure 5C).

**FIGURE 6:**
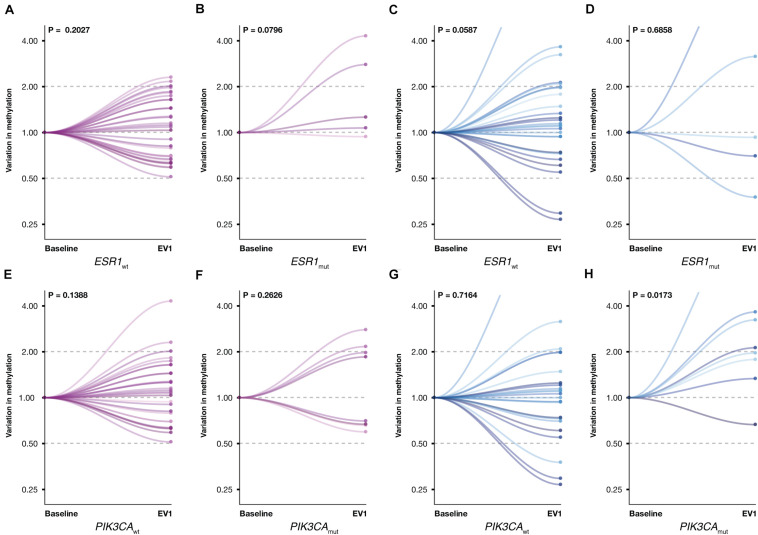
Promoter A (purple) and B (blue) variations between BL and EV1 according to *ESR1* and *PIK3CA* mutational status. A significant increase was observed for promB in PIK3CA-mutated patients **(H)**. A numerical difference was observed for promA in the *ESR1*-mutated subgroup **(B)** and promB in ESR1 wild-type MBC **(C)**. No significant promA variations were observed in ESR1 wild-type patients **(A)** or with respect to PIK3CA mutational status **(E,F)**. No significant promB variations were observed in ESR1-mutated or in PIK3CA wild-type patients **(D,G)**.

Given the established role of *ESR1* and *PIK3CA* gene mutations in ET resistance, promA and promB variations were investigated according to *ESR1* and *PIK3CA* mutational status (Figure 6). A significant increase was observed for promB among patients with *PIK3CA* mutation (*P* = 0.0173) (Figure 6H). A trend was observed for promA in the *ESR1* mutant subgroup (Figure 4B) and for promB in *ESR1* wild-type patients (Figure 6C).

## Discussion

*De novo* and acquired ET resistance result in cancer progression and metastatic spreading, limiting clinical benefit in luminal-like MBC. Mutations of the *ESR1* gene have been described in 20% of ET-resistant cases and represents one of the main ET resistance mechanisms, but the relationship between epigenetic events and treatment-induced resistance is still unclear (10, 27–30).

The present study tested the feasibility of evaluating *ESR1* CRE hypermethylation in liquid biopsies from luminal-like MBC and explored the potential impact on prognosis of its dynamics.

The most transcriptionally active *ESR1* promoters (promoter A and B) were analyzed at baseline and after 3 months of ET. No significant cross-associations were observed between the two, suggesting their independent role and potential for mutual integration. The baseline levels of promA were associated with metastatic liver involvement, which was confirmed also on multivariate analysis. Uncovering the link between metastatic behavior and tumor biology is an unmet clinical need as it can potentially enable a more granular prognostic stratification and guide disease monitoring strategies by suggesting the best imaging technique for each patient (25, 31–33).

The prognostic role of promA and promB was moreover explored. Although no prognostic impact was observed for both BL promA and promB, a 2-fold increase in promB and in either promA and promB after 3 months of ET was associated with a worse PFS and OS. Notably, PFS results were consistent across clinically relevant subgroups, such as ET backbone and *ESR1* and *PIK3CA* mutational status. Although preliminary, these results may suggest the added value of longitudinal genetic and epigenetic monitoring as the prognostic role of *ESR1*-associated features is likely the result of a complex interplay between inherit biological features and acquired resistance mechanisms induced by ET, rather than a baseline characteristic.

A similar scenario was described by the preliminary results of the PADA-1 study which explored the impact of baseline *ESR1* mutation and its dynamics on PFS. Out of the 32 analyzed patients, 25 (78%) had an *ESR1* mutation that became undetectable in ctDNA within the first 5 months of ET. Although 14 patients had a recurrence of *ESR1* mutation with a consequent progression, 9 patients (36%) were still free from *ESR1* mutation and progression at the time of analysis. Intriguingly, 2 patients (8%) experienced a progression without the recurrence of *ESR1* mutation. Since the study was based on a genetic-only characterization, these results suggest the need for a wider, genetic, and epigenetic characterization to better characterize the complexity of ET resistance (34).

It has previously been shown that *ESR1* promoter methylation was significantly associated with either ER-negative early BC or with a heterogenous ER expression in the luminal-like disease (27). This suggested that silencing by methylation could affect the expression of ER and in turn treatment response, although without evidence of a survival impact (27).

Endocrine-resistant cells are characterized by a distinctive DNA methylome. Moreover, hypermethylation can affect estrogen-regulated enhancers resulting in a reduced *ESR1* binding with a consequent decreased gene expression of key effectors of *ESR1* activity (12). Furthermore, *ESR1*-responsive enhancer hypomethylation is critical in the transition from normal epithelial cells to endocrine-responsive luminal-like BC, supporting the concept that dynamic epigenetic variations are pivotal in cancer evolution (12).

Notwithstanding the preliminary follow-up and sample size, our results suggest that the combination of promA and promB dynamics could be more informative than the *ESR1* mutational status alone, since epigenetic modifications have a faster onset and could realistically be able to grasp features linked to an inherently lower sensitivity to ET (10, 28–30).

The added value of a longitudinal strategy is also supported by the lack of impact on PFS of baseline promA and promB. If on one hand mutational alterations of *ESR1* are late events mainly induced by therapy and often maintained across the subsequent lines, on the other, epigenetic alterations are highly dynamic and reflect the actual real-time changes caused by ET at the moment of the blood draw.

Alternative approaches for a more comprehensive liquid biopsy-based characterization of luminal-like MBC were initially focused on CTCs. A previously reported pilot study based on 17 patients after progression to ET (respectively 7 treated with fulvestrant and 10 with AI) highlighted an association between ET resistance and both enumeration and heterogeneous ER expression of CTCs (35). Notably, all patients treated with AI showed a heterogeneous ER expression in CTCs, while two patients treated with fulvestrant had no detectable CTC expression of ER and three had heterogeneous CTC ER, probably due to an incomplete degradation of the ER target by fulvestrant (35). A larger study characterized 122 MBC patients through a methylation-specific (MS) qPCR assay applied to CTCs and, in a smaller subgroup (58 patients), in matched plasma samples. *ESR1* methylation was observed in 10 out of 36 CTC-positive samples (27.8%), was highly concordant with the paired ctDNA samples, and was moreover associated with a lack of response to everolimus and exemestane (22). These results, although supporting the concept of integrated genetic and epigenetic events concurring to ET resistance, are on the other hand limited to patients defined as Stage IV_*aggressive*_ through the CellSearch platform (i.e., with ≥5 CTCs/7 ml of blood), a population limited to 45% of the overall MBC patients and up to 49% in the luminal-like subgroup (36).

A ctDNA approach, on the other hand, can be applied to a broader cohort of patients also thanks to the higher sensitivity of ddPCR.

In our cohort, *ESR1* and *PIK3CA* mutations were detected, respectively in 14 and 23% of patients and, intriguingly, a significantly lower promA was highlighted in patients with *ESR1* mutation.

*ESR1* is often affected by activating mutations, such as Y537 and D538 (located in the helix 12 of *ESR1* ligand binding domain), which result in an agonist form of *ESR1* (28). Given these premises, it is mechanistically tempting to think that promoter methylation could represent a detrimental event to an acquired resistance advantage and that the latter could be hindered by epigenetic silencing.

Although intriguing and prospectively generated, these results may have some limitations. If on one hand the proof of concept study enrolled a homogeneous cohort of luminal MBC patients treated with first-line ET and was therefore an appropriate basis for hypothesis generation and for testing the technological transferability of longitudinal epigenetic characterization, on the other hand, the sample size is limited and hinders a deeper characterization of subgroups of particular interest. Consistently, the median follow-up is limited and may underestimate the real prognostic impact of the analyzed biomarkers. Moreover, additional timepoints could increase promA and promB curve’s resolution and therefore provide precious information for optimal timing definition.

## Conclusion

The present study proofed the concept that an epigenetic characterization strategy based on ctDNA can be integrated in the current clinical workflow of patients with metastatic breast cancer to give useful insights on treatment sensitivity. Although exploratory, these results support the emerging new concept of a dynamic epigenetic profiling. Based on these data, a cohort expansion is planned to validate and further develop this approach.

## Data Availability Statement

The original contributions presented in the study are included in the article/Supplementary Material. Some datasets presented in this article are not readily available because are part of an ongoing clinical trial. Requests to access the datasets should be directed to corresponding author.

## Ethics Statement

The studies involving human participants were reviewed and approved by the Comitato Etico Unico Regionale del Friuli Venezia Giulia. The patients/participants provided their written informed consent to participate in this study. Written informed consent was obtained from the individual(s) for the publication of any potentially identifiable images or data included in this article.

## Author Contributions

LG, DB, MB, and FP conceived the study, participated in its design and coordination, recruited the patients, created and updated the clinical database, performed the statistical analysis, and wrote the manuscript. AF, LA, and GD optimized the preanalytical and extraction workflow and the MS-ddPCR assay, and helped to draft the manuscript. DV, BB, and GB optimized and performed the ctDNA NGS analysis and helped to draft the manuscript. CC, LB, EB, SB, GT, LD, and SR recruited the patients and updated the clinical database. All authors read and approved the final manuscript.

## Conflict of Interest

LG has received reimbursement for travel expenses from Menarini Silicon Biosystems. FP received research funding from AstraZeneca. The remaining authors declare that the research was conducted in the absence of any commercial or financial relationships that could be construed as a potential conflict of interest.

## Supplementary Material

TABLE S1 ESR1 and PIK3CA mutational status for all patients enrolled in the CRO-2018-56 study.
