# Supplementary material for: Plasma-Based Longitudinal Evaluation of ESR1 Epigenetic Status in Hormone Receptor-Positive HER2-Negative Metastatic Breast Cancer
Source: Front Oncol. 2020 Sep 18;10:550185. doi: 10.3389/fonc.2020.550185 (PMC7531252; doi:10.3389/fonc.2020.550185)
Supplement: TABLE S1 — ESR1 and PIK3CA mutational status for all patients enrolled in the CRO-2018-56 study. [file Table_1.docx]

| **ID** | **PIK3CA** | **ESR1** |
| --- | --- | --- |
| 1 | wt | wt |
| 2 | wt | Y537S 1.4% |
| 3 | H1047R 24.6% | wt |
| 4 | wt | wt |
| 5 | H1047R 1.2% | wt |
| 6 | wt | wt |
| 7 | wt | wt |
| 8 | wt | wt |
| 9 | E545K 1.2% | wt |
| 10 | wt | Y537S 7.4% |
| 11 | wt | wt |
| 12 | wt | D538G 1.5% |
| 13 | H1047R 39.1% | Y537S 1.9% |
| 14 | wt | wt |
| 15 | wt | wt |
| 16 | wt | wt |
| 17 | H1047R 3.1% | wt |
| 18 | H1047R 10.3% | wt |
| 19 | wt | wt |
| 20 | wt | wt |
| 21 | H1047L 6.6% | wt |
| 22 | wt | Y537N 7.3% |
| 23 | wt | wt |
| 24 | wt | wt |
| 25 | wt | wt |
| 26 | wt | wt |
| 27 | wt | wt |
| 28 | wt | wt |
| 29 | wt | wt |
| 30 | wt | H377R 14% |
| 31 | H1047R 10.1% | wt |
| 32 | H1047R 1.2% | wt |
| 33 | wt | wt |
| 34 | wt | wt |
| 35 | E545K 1% | wt |
| 36 | wt | D538G 33.7% |
| 37 | n/a | n/a |
| 38 | wt | wt |
| 39 | n/a | n/a |
| 40 | H1047R 16.9% | wt |
| 41 | wt | wt |
| 42 | wt | wt |
| 43 | wt | wt |
| 44 | wt | wt |
| 45 | wt | wt |
| 46 | wt | wt |
| 47 | wt | wt |
| 48 | wt | wt |
| 49 | wt | wt |

**Supplemental Table 1.** ESR1 and PIK3CA mutational status for all patients enrolled in the CRO-2018-56 study
